# Development of 3D Food Printing Ink Based on Germinated Brown Rice Modified by Dry Heat and High-Pressure Steam

**DOI:** 10.3390/foods15203693

**Published:** 2026-10-17

**Authors:** Yuxiang Shen, Jinglei Xu, Zhongbo Zhang, Jiping Wen

**Affiliations:** College of Food Science and Engineering, Henan University of Technology, 100 Lianhua Street, High-Tech, Zhengzhou 450001, China; m18336399853@163.com (Y.S.); xu2239942856@163.com (J.X.); zhangzb231@163.com (Z.Z.)

**Keywords:** 3D printing, germinated brown rice, dry-heat treatment, high-pressure-steam treatment, rheological properties

## Abstract

High-performance whole-grain ingredients that integrate superior nutritional properties and favorable printability are essential for the advancement of 3D food printing technology. Germinated brown rice (GBR) is rich in γ-aminobutyric acid (GABA), a key bioactive compound, rendering it a viable whole-grain material for 3D food printing applications. Nevertheless, native GBR-based gels suffer from poor dimensional accuracy during 3D printing, which severely restricts their practical application. To tackle this issue, this study adopted a green heat treatment method to improve both the nutritional benefits and 3D printing performance of GBR. Brown rice was first germinated to obtain GBR, followed by modification via dry heat treatment (90–150 °C) or high-pressure steam treatment (5–25 min). The effects of the two treatments on GABA retention, starch recrosslinking, gel rheology, texture, microstructure, and printing accuracy were systematically characterized. Results indicated that germination significantly elevated GABA content from 47.66 mg/kg in brown rice to 104.85 mg/kg in GBR, but unmodified GBR gels exhibited relative dimensional deviations of 9.01% and 18.70% for the “letter W” and “Android robot” models, respectively. Moderate thermal modification retained and further improved GABA bioactivity, while promoting starch recrosslinking to form a uniform porous gel network. The optimal groups (GD-135 and GS-15) yielded significantly reduced printing deviations (*p* < 0.05), with a maximum deviation reduction exceeding 93%. Collectively, dry heat and high-pressure steam modification enabled concurrent improvement of GABA retention and 3D printing precision of GBR. This work provides a non-chemical, energy-efficient green modification approach for whole-grain raw materials applied in personalized 3D-printed foods.

## 1. Introduction

As a transformative digital manufacturing technology, 3D food printing produces customized three-dimensional food structures based on computer-aided design (CAD) modeling and precise layer-by-layer deposition of edible materials [1,2,3]. This technology offers substantial economic benefits by reducing waste, enables on-site food production at diverse locations, and increasing food supply. Modified GBR inks can make full use of underdeveloped whole grain resources, meeting people’s increasingly personalized nutritional needs. This reinforces the prospects outlined above. Within the context of sustainable agricultural food systems and a circular economy, 3D food printing can precisely control ingredient amounts, minimize whole grain waste, and reduce carbon emissions from long-distance transport and centralized processing [4,5,6]. From a more personal perspective, 3D food printing can precisely control the texture and nutrition of food, which is especially helpful for the elderly or people with swallowing difficulties and is also key to improving public nutrition and overall health [7,8,9].

Rice is an important staple crop worldwide. Brown rice (BR) retains the bran and germ after the husk is removed, and compared to refined white rice, it is rich in dietary fiber, minerals, GABA, ferulic acid, and other bioactive compounds [10]. However, most of its nutrients are not fully released, and issues such as the tough texture and rough mouthfeel of the bran layer limit its direct consumption and further processing [11]. GBR, made by controlled-temperature germination [12], uses endogenous enzymes to break down large polymers, enriching functional components such as phenolics, tocopherols, and GABA [13,14]. It has clear benefits for improving metabolic syndrome, enhancing neuroprotection [15], and regulating gut microbiota, making it a more nutritious whole-grain ingredient. Its gelatinization and rheological properties are also different from regular brown rice [12]. Even so, the coarse fiber still induces a gritty texture, so it is often made into powders to improve eating experience and storage. GBR is high in starch with excellent gelling properties, making it a promising whole-grain material for food 3D printing [15]. However, most existing research on GBR focuses on germination process optimization and nutrition enhancement, with little study on its 3D printability.

Green physical modification has become the main method for adjusting cereal raw materials because it does not leave chemical residues, is relatively easy to operate, and has great potential for industrial scale-up [16]. Heat treatment is a green physical modification technique that can induce starch gelatinization and protein denaturation, and regulate intermolecular forces, changing powder particles, rheology, and hydration properties. It is an effective way to improve cereal printing performance. Qin et al. [17] found that dry heat can strengthen the interaction between starch and protein molecules in glutinous rice flour, enhancing gel viscoelasticity and helping the paste flow smoothly through the nozzle. Kou et al. [7] showed that samples pretreated at 200 °C had the best printing accuracy, and adjusting the filling rate can produce 3D instant highland barley foods that meet dysphagia standards. Lee et al. [18] pointed out that high-pressure steam treatment disrupts the microscopic structure of wheat bran, increases soluble dietary fiber and total phenol content, and enhances WHC. Chen et al. [19] indicated that thermal treatments such as baking and extrusion puffing can break starch crystalline structures and reshape volatile flavors. Nevertheless, to date, most studies on heat treatment of grain-based foods have mainly looked at glutinous rice flour, wheat starch, and regular brown rice. In contrast, relevant research on GBR remains limited. Specifically, no systematic comparisons between dry heat and high-pressure steam treatments have been reported concerning starch multi-scale structure, GABA retention capacity, and 3D printability of GBR. Furthermore, the structure–function relationship between thermally induced starch structural alterations and the printing accuracy of GBR gels remains poorly elucidated.

To bridge this research gap, this study prepared GBR from brown rice and modified it via dry heat treatment or high-pressure steam treatment. The effects of the two thermal modification methods on GBR bioactivity, physicochemical properties, gel rheology, texture, microstructure, and 3D printing performance were systematically investigated. The objectives of this study were to clarify the regulatory effect of thermal modification on GBR gel structure and printing performance, identify the optimal modification parameters, and reveal the structure–function relationship between multi-scale structural changes in starch and printing fidelity. This study provides a theoretical foundation and technical guidance for the development of high-nutrient whole-grain 3D-printing inks, and offers a green and efficient modification pathway for the high-value utilization of germinated brown rice, thereby promoting the industrial application of whole-grain 3D-printed foods and supporting the sustainable development of the agri-food processing industry.

## 2. Materials and Methods

### 2.1. Materials and Chemicals

Brown rice was purchased from Heilongjiang Cangtian Natural Ecological Agriculture Co., Ltd. (Harbin, China). γ-Aminobutyric acid (GABA, purity ≥ 99%) and other chemicals and solvents were supplied by Shanghai Yuanye Bio-Technology Co., Ltd. (Shanghai, China) and Sigma-Aldrich (St. Louis, MO, USA), respectively. All reagents were of analytical grade unless specified otherwise.

### 2.2. Sample Preparation

#### 2.2.1. Preparation of Germinated Brown Rice Powder

BR was sorted and washed repeatedly with deionized water. The cleaned brown rice was soaked in deionized water at 30 °C for 12 h and then evenly spread on gauze-lined trays. Germination was conducted in a constant temperature and humidity incubator at 30 °C and ≥95% relative humidity (RH) for 24 h. Prior to drying, the average sprout length reached approximately 35% of the grain length. The samples were subsequently dried in a hot-air oven at 45 °C for 8 h to a final moisture content of 9.25%, ground into powder, and passed through a 90-mesh sieve. The resultant powder was packed in self-sealing bags and stored at −20 °C until use.

For the dry heat treatment, germinated brown rice powder was spread evenly on baking trays and treated in a pre-heated drying oven for 20 min at 90 °C, 105 °C, 120 °C, 135 °C and 150 °C, respectively. The dry heat-treated germinated brown rice powder samples were designated as GD-90, GD-105, GD-120, GD-135 and GD-150. For the high-pressure steam treatment, germinated brown rice powder was spread on the bottom of Erlenmeyer flasks, and heat-treated using an autoclave at 115 °C for 5, 10, 15, 20 and 25 min. After cooling to room temperature, the moisture of the treated powder was continuously adjusted by drying and repeatedly measured to approach the initial moisture level of GBR. The moisture was considered to reach the target level when the difference between the measured value and the target moisture was ≤0.5%. The corresponding samples were named GS-5, GS-10, GS-15, GS-20 and GS-25, respectively. BR and GBR were used as the control groups.

#### 2.2.2. Preparation of Germinated Brown Rice 3D-Printing Ink

The GBR powder was dispersed in deionized water to prepare a 25% (*w*/*w*) suspension. The suspension was heated in a water bath at 80 °C under constant stirring at 60 rpm for 10 min to achieve complete gelatinization. Subsequently, the gel was removed from the water bath and allowed to cool to room temperature for 15 min, then reserved for subsequent use.

### 2.3. 3D Food Printing

The prepared gel was transferred into the syringe of a FOODBOT-S2PRO food 3D printer (Shiyin Technology Co., Ltd., Hangzhou, China) to evaluate its printability. Printing experiments were performed at ambient temperature (ca. 25 °C). The key printing parameters were optimized through preliminary trials specifically tailored to the rheological characteristics of GBR gel systems and ultimately established as follows: nozzle inner diameter 0.84 mm, printing temperature 25 °C, and nozzle travel speed 70 mm/s. Two geometric models, namely a “W-shaped” model (25 mm × 35 mm × 5 mm) and an Android robot model (26 mm × 25 mm × 34 mm), were printed. Upon completion of printing, the actual dimensions of printed constructs were measured. Material printability was assessed based on the morphological features and dimensional accuracy of printed samples. The relative dimensional deviation was calculated using Formulas (1) and (2):

(1) $$E_{x}=\frac{\left|S_{x}-M_{x}\right|}{M_{x}}\times 100,\;\;\;\;\;x\in \{L,D,H\}$$

(2) $$E_{a}=\frac{E_{L}+E_{D}+E_{H}}{3}$$

where *E_x_* is the relative deviation for each dimension of the printed sample (*x* denotes length *L*, width *D*, height *H*), *S_x_* is the measured dimension of the printed sample, *M_x_* is the theoretical dimension of the model, *E_L_*, *E_D_*, *E_H_* are the relative deviations of length, width and height, respectively, and *E_a_* is the average relative deviation of the sample.

### 2.4. GABA Content

The GABA content in thermally treated GBR powder was measured following the method described by Luo et al. [10] with minor modifications. Briefly, 0.1 g of GBR powder was accurately weighed and ultrasonically extracted with deionized water in three consecutive cycles. After centrifugation, the resulting supernatants were combined. Subsequently, 200 μL of 2.5% (*v*/*v*) phenyl isothiocyanate (PITC) in acetonitrile and 400 μL of triethylamine-acetonitrile mixture (1.4:8.6, *v*/*v*) were added in sequence. The reaction mixture was kept at room temperature for 2 h, after which 1 mL of n-hexane was added and the solution was vortex-mixed. After separating the phases, the lower aqueous layer was collected, filtered through a 0.45 μm membrane, and dried with nitrogen. Then, we dissolved the residue in a 50% methanol–water solution for the next HPLC test.

Chromatographic separation was performed using a GOLD C18 column (4.6 mm × 250 mm, 5 μm). The mobile phases were solution A (4.1 g/L sodium acetate buffer with acetonitrile, 95:5 by volume) and solution B (acetonitrile). The gradient elution program was set as follows: 0–15 min, acetonitrile increased from 5% to 10%; 15–20 min, it increased to 100%. We detected at 254 nm, used a 1.0 mL/min flow rate, and kept the column at 30 °C. We measured the GABA concentration using an external standard curve.

### 2.5. Water-Holding (WHC) and Oil-Holding Capacity (OHC)

WHC and OHC of GBR flour were measured following Dong et al. [20], with minor modifications. For WHC, we made a 5% (*w*/*w*) GBR flour mix in deionized water; for OHC, we made the same kind of mix but with sunflower oil. After mixing it evenly, we let the suspension sit at room temperature for 1 h, then put it in a centrifuge and spin at 4000 rpm for 20 min. We poured off the supernatant and inverted it on filter paper, then finally weighed the sediment.

The WHC and OHC were then calculated using Formulas (3) and (4):

(3) $$\mathrm{WHC}(g/g)=\frac{m_{1}-m_{0}}{m_{0}}$$

(4) $$\mathrm{OHC}(g/g)=\frac{M_{1}-M_{0}}{M_{0}}$$

where *m*_0_ is the dry mass of the sample, *m*_1_ is the wet mass of the sample after centrifugation, *M*_0_ is the dry mass of the sample before oil absorption, and *M*_1_ is the oil-absorbed wet mass of the sample after centrifugation.

### 2.6. Pasting Properties

Referring to the method of Cheng et al. [21] with some adjustments, we used a Rapid Visco Analyzer (RVA, Newport Scientific, Warriewood, Australia) to measure the pasting properties of the samples. Briefly, we poured 25 mL of distilled water into the RVA sample canister, then accurately weighed 3 g of germinated brown rice flour and added it to the canister. We then placed the canister into the RVA for measurement. The recorded data include peak viscosity (PV), trough viscosity (TV), breakdown (BD), final viscosity (FV), setback (SB), peak time (PT), and pasting temperature (Ptemp).

### 2.7. Rheological Measurements

Dynamic rheological properties of gel samples were determined using a MARS60 rheometer (Thermo Fisher Scientific, St. Louis, MO, USA) following Zheng et al. [2] with minor modifications. Briefly, the gel sample was promptly transferred onto the rheometer measuring plate for dynamic frequency-sweep tests to characterize its viscoelastic behaviour. A 35 mm cone-plate geometry was used with a gap set at 1 mm, and the measuring temperature was kept constant at 25 °C. Oscillatory measurements were carried out within the linear viscoelastic region under a fixed strain of 10%, with the angular frequency ranging from 0.1 Hz to 10 Hz.

### 2.8. Texture Profile Analysis (TPA)

TPA of germinated brown rice gel was performed using a TA-XT Plus texture analyzer (Stable Micro Systems, Godalming, UK). Prepared gel samples were conditioned at 4 °C for 24 h before measurement. A P/0.5R cylindrical probe was used with a trigger force of 5 g. Pre-test, test, and post-test speeds were uniformly set to 1 mm/s, with a compression ratio of 30%. The resting period between two compression cycles was 3 s. All TPA parameters were recorded during the test.

### 2.9. Low-Field Nuclear Magnetic Resonance (LF-NMR) Moisture Determination

The moisture distribution of GBR gel was characterized using a VAMR20-010V-T variable-temperature LF-NMR analyzer (Niumag Electric Corporation, Shanghai, China) following Cui et al. [22] with minor modifications. Briefly, 0.6 g of gel sample was accurately weighed and transferred into a plastic tube, which was sealed with Parafilm to prevent moisture loss throughout measurement. The sealed plastic tube was placed inside a standard NMR sample tube for LF-NMR acquisition. The instrumental parameters were set as follows: number of scans (NS) = 4; number of echoes = 3000; 90° pulse width (P1) = 6.52 μs; 180° pulse width (P2) = 12 μs; time domain (TD) = 240018; spectral width (SW) = 100 kHz; waiting time (TW) = 4000 ms.

### 2.10. Fourier Transform Infrared (FTIR) Spectroscopy Analysis

Sample powder (1 mg) and potassium bromide (KBr, 100 mg) were accurately weighed, finely ground, and homogeneously blended in an agate mortar. The powder mixture was subsequently pressed into transparent KBr pellets. FTIR spectra were acquired using a Spectrum TWO spectrometer (PerkinElmer, Shelton, CT, USA) in transmission mode. Spectra were collected over the wavenumber range of 4000–400 cm^−1^ with 32 scans, under a controlled temperature of (20 ± 0.2) °C.

### 2.11. X-Ray Diffraction (XRD) Analysis

XRD patterns were collected using a Rigaku SmartLab SE X-ray diffractometer (Rigaku Corporation, Osaka, Japan). The instrument was operated at 40 kV and 40 mA, with a scanning rate of 10°/min over a 2θ range of 5–80° [23].

### 2.12. Scanning Electron Microscopy (SEM)

The microstructures of germinated brown rice flour particles and freeze-dried cross-sections of 3D-printed samples were observed by SEM. Samples were fixed onto aluminum stubs with double-sided conductive tape and sputter-coated with a thin gold layer. Micrographs were obtained using a ZEISS Sigma 300 scanning electron microscope (ZEISS, Oberkochen, Germany) under an accelerating voltage of 10 kV. Representative images were acquired at magnifications of ×200 and ×3000.

### 2.13. Statistical Analysis

All experiments were carried out in triplicate, and quantitative results are presented as mean ± standard deviation (SD). Statistical analysis was performed using SPSS Statistics 24.0 (IBM Corp., Armonk, NY, USA). One-way analysis of variance (ANOVA) coupled with Duncan’s multiple-range test was used to evaluate inter-group differences. Datasets from dry heat treatment and high-pressure steam treatment were analyzed separately. A significance level of *p* < 0.05 was considered statistically significant. All figures were generated using Origin 2019b (OriginLab Corp., Northampton, MA, USA).

## 3. Results and Discussion

### 3.1. 3D Food Printing Performance

**Figure 1:**
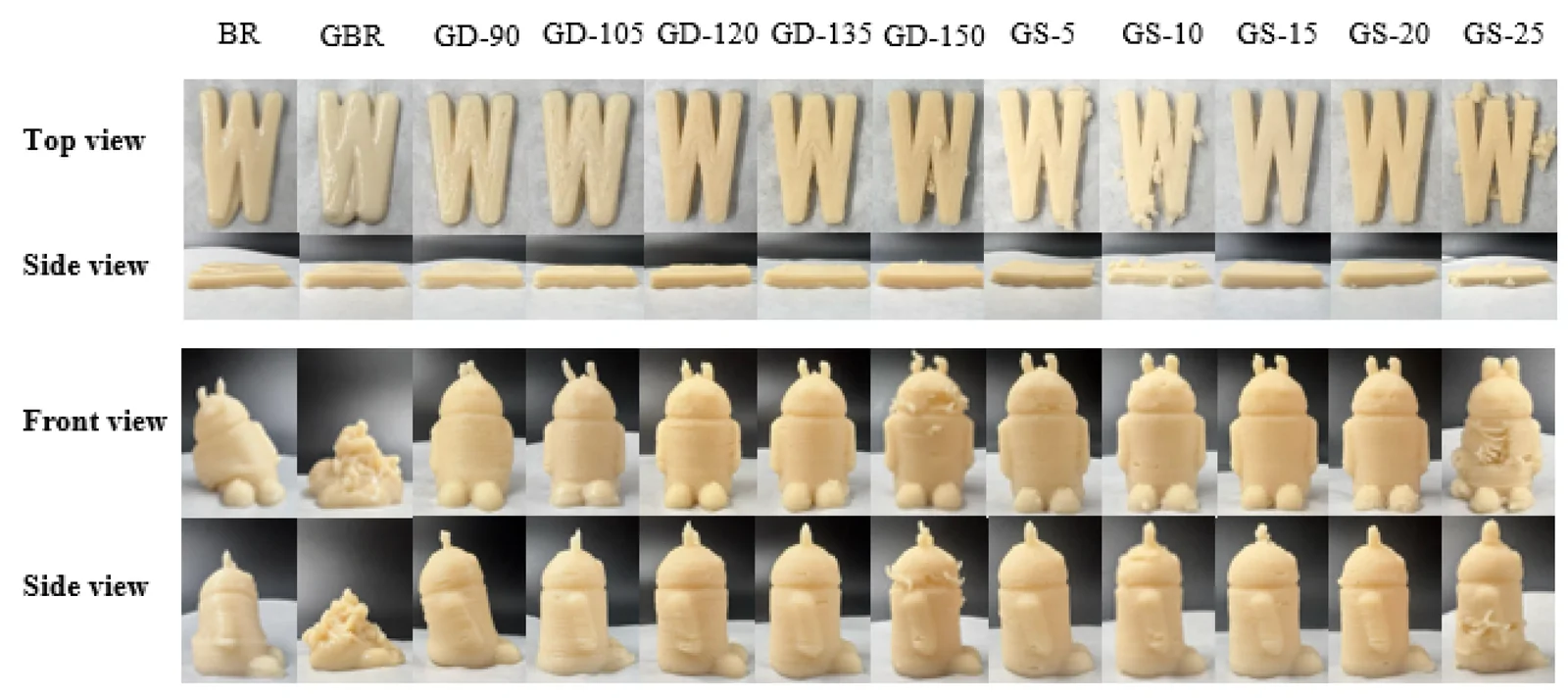
Macroscopic morphology of 3D-printed samples fabricated from gels of dry heat-treated and high-pressure steam-treated germinated brown rice.

**Figure 2:**
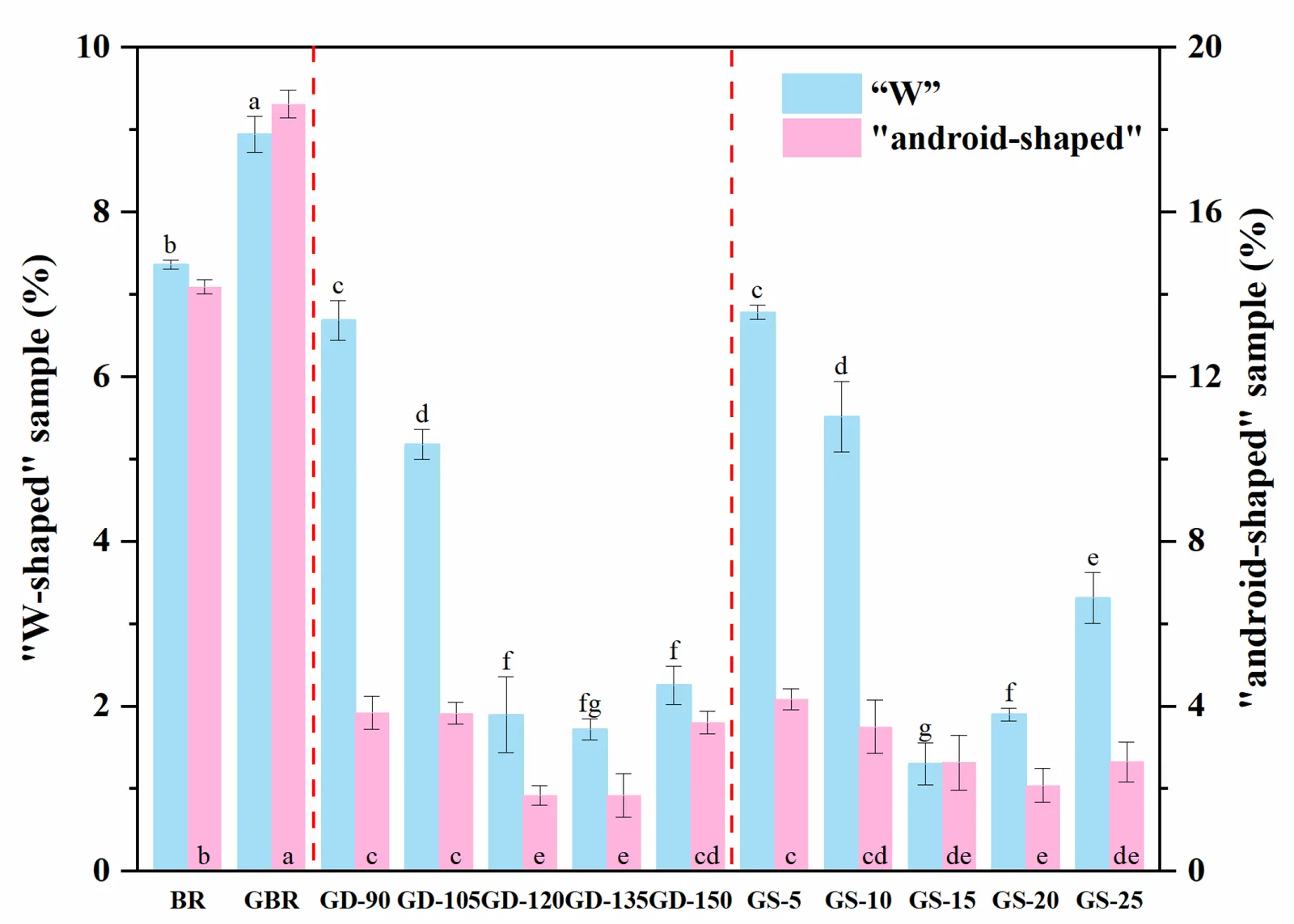
Relative deviation of 3D-printed W-shaped and Android-shaped models fabricated from gels of dry heat-treated and high-pressure steam-treated germinated brown rice. Left Y-axis: relative deviation of W-shaped model; right Y-axis: relative deviation of Android-shaped model. Different lowercase letters indicate significant differences (*p* < 0.05).

The intact contour, distinct layers, clear surface texture and structural support capacity of 3D-printed samples serve as core indicators for evaluating printing quality [21]. The printing performance is closely associated with gelatinization properties, dynamic rheological behaviors and textural characteristics of gels [22]. In this study, two printing models, the planar “W” and the three-dimensional “android robot”, were adopted to investigate the effects of dry heat and high-pressure steam treatments on the 3D-printing performance of gels prepared from germinated brown rice flour. The macroscopic morphologies of printed samples were shown in Figure 1, and their dimensional relative deviations were presented in Figure 2.

Dry heat treatment effectively improved the printing performance of germinated brown rice gels. With increasing treatment temperature, the dimensional deviations of both models decreased first and then increased. The GD-135 group achieved the minimum dimensional deviation and optimal printing accuracy. For groups from GD-90 to GD-135, the planar model possessed sharp edges and continuous lines, and the three-dimensional model exhibited clear contours with tight inter-layer deposition without collapse. Appropriate dry heat treatment optimized gel texture, enhanced the compactness of gel network and water-binding capacity, and thus endowed printing ink with favorable extrudability as well as post-extrusion shape-holding capacity. When the temperature rose to 150 °C, burrs and filament breakage appeared on sample edges, and details of the three-dimensional model were damaged. This was mainly caused by excessive gel hardness and deteriorated fluidity, which impaired extrusion uniformity. High-pressure steam treatment also optimized the printing performance of germinated brown rice gels. As treatment time increased, dimensional deviations decreased first and then increased. Both GS-15 and GS-20 groups maintained low dimensional deviations for the two models. Moderate high-pressure steam treatment promoted sufficient starch gelatinization to form a uniform and fine gel network, yielding favorable surface smoothness for planar printed models. Nevertheless, owing to the high-moisture condition of hydrothermal system, the improvement in overall gel strength was limited, so that the structural support for three-dimensional constructs was inferior to that of dry heat-treated samples. Excessively long treatment time triggered excessive starch degradation and further impaired the formability of gels.

In conclusion, both moderate dry heat and high-pressure steam treatments enhanced the 3D printing performance of germinated brown rice gels. Dry heat treatment exerted a more prominent effect on improving structural support for three-dimensional constructs, while high-pressure steam treatment contributed to superior printing accuracy for planar models. Considering both macroscopic appearance and dimensional deviation results, germinated brown rice gels treated by GD-135 dry heat and GS-15 high-pressure steam possessed the optimal 3D-printing adaptability, which provided a reference for raw-material modification of germinated-brown-rice-based 3D-printed foods.

### 3.2. Variations in GABA Content

**Figure 3:**
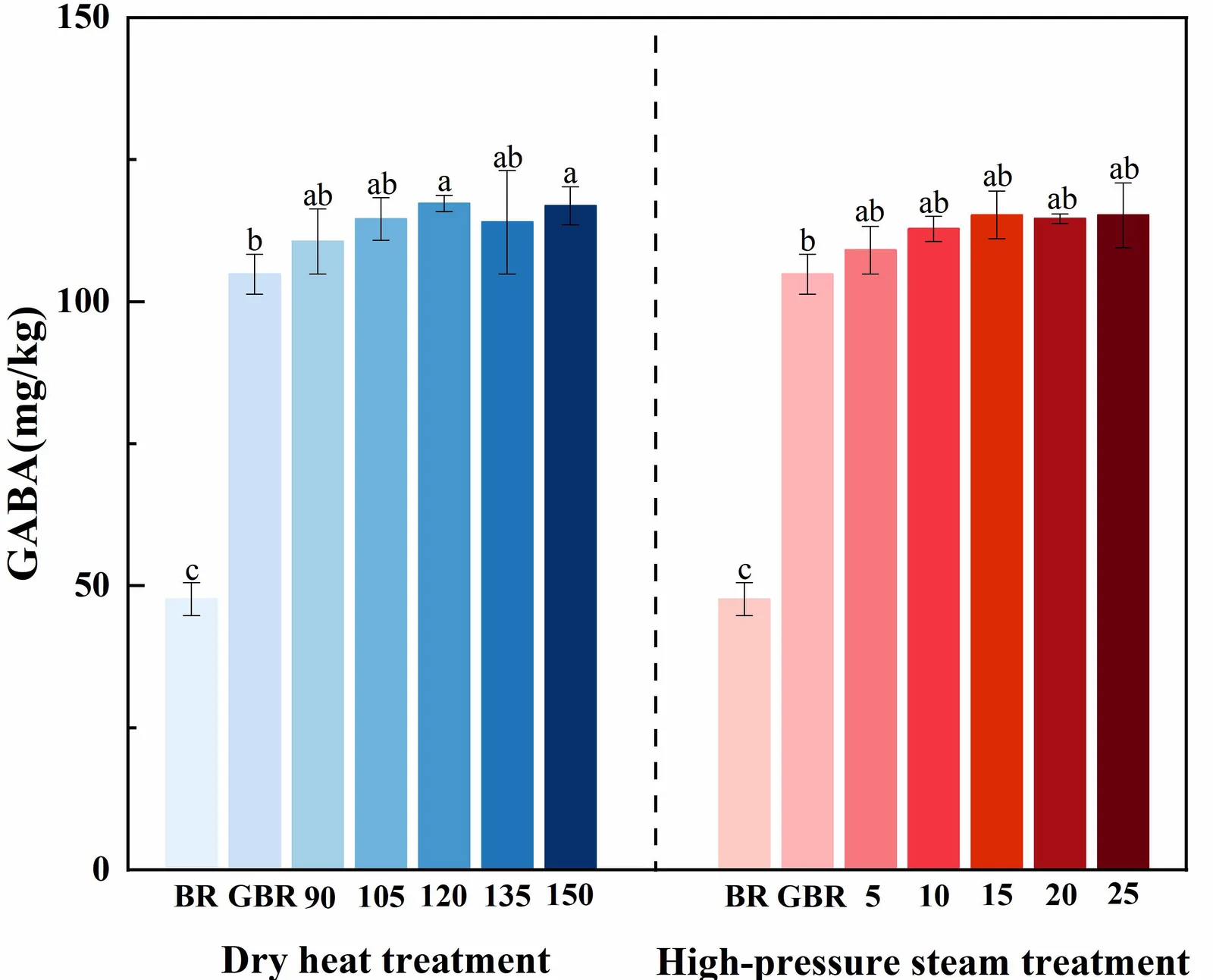
GABA content of dry heat-treated and high-pressure steam-treated germinated brown rice flour. Different lowercase letters indicate significant differences among groups (*p* < 0.05).

GABA acts as an inhibitory neurotransmitter in the central nervous system and exhibits multiple physiological functions including blood-pressure-lowering, anxiolytic, neuroprotective and antioxidant activities [13]. As illustrated in Figure 3, BR contained a relatively low level of GABA. Germination markedly elevated GABA content from 47.66 mg/kg to 104.85 mg/kg *(p* < 0.05), corresponding to a 119.95% increment. On this basis, the present study further explored how dry heat treatment and high-pressure steam treatment modulated GABA accumulation in GBR.

In the dry heat treatment group, GABA content presented an overall plateau trend with an initial increase followed by a gradual decline as the treatment temperature elevated. The GD-120 sample achieved the maximum GABA content of 117.29 mg/kg, which was 146.05% and 11.86% higher than that of raw BR and untreated GBR, respectively. Notably, although GD-120 possessed the highest nutritional value, GD-135 was determined as the optimal treatment condition considering the comprehensive balance of nutritional retention and 3D printability. Compared with GD-120, the GD-135 sample exhibited a slight GABA reduction of 2.78% (114.02 mg/kg), while its printing accuracy was significantly improved. For example, the relative size deviation of the W-type model decreased from 1.90% to 1.72%, and the Android robot model dropped from 1.26% to 0.93%. Compared to native germinated brown rice (which had deviations of 9.01% and 18.70%), GD-135’s dimensional errors decreased by 80.9% and 95.0%. For the high-pressure steam treatment group, the GABA content increased as the treatment time increased. Unlike dry heat treatment, GS-15 showed the best GABA retention and favorable printability among all the steam-treated samples, achieving an excellent balance between nutritional performance and processability.

Based on existing research, during the germination process, the endogenous glutamate decarboxylase (GAD) is activated, catalyzing the conversion of glutamate into GABA, which makes the baseline GABA in GBR much higher than in BR [24]. Moderate heat treatment can loosen starch gels and break cell membranes, releasing pre-synthesized GABA entrapped inside the granules and tissue matrix, leading to a moderate rise in detectable GABA content. However, excessively high temperature or prolonged holding time can cause thermal degradation of free GABA, limiting further accumulation. To compare the two heat treatment methods, both dry heat and high-temperature high-pressure steaming can, under suitable process conditions, preserve or even slightly increase the GABA level in germinated brown rice, making them effective processing methods for regulating the functional components of germinated brown rice, enhancing the nutritional quality and processing suitability of brown rice, and endowing the material with great potential to improve its added value.

### 3.3. WHC and OHC

**Figure 4:**
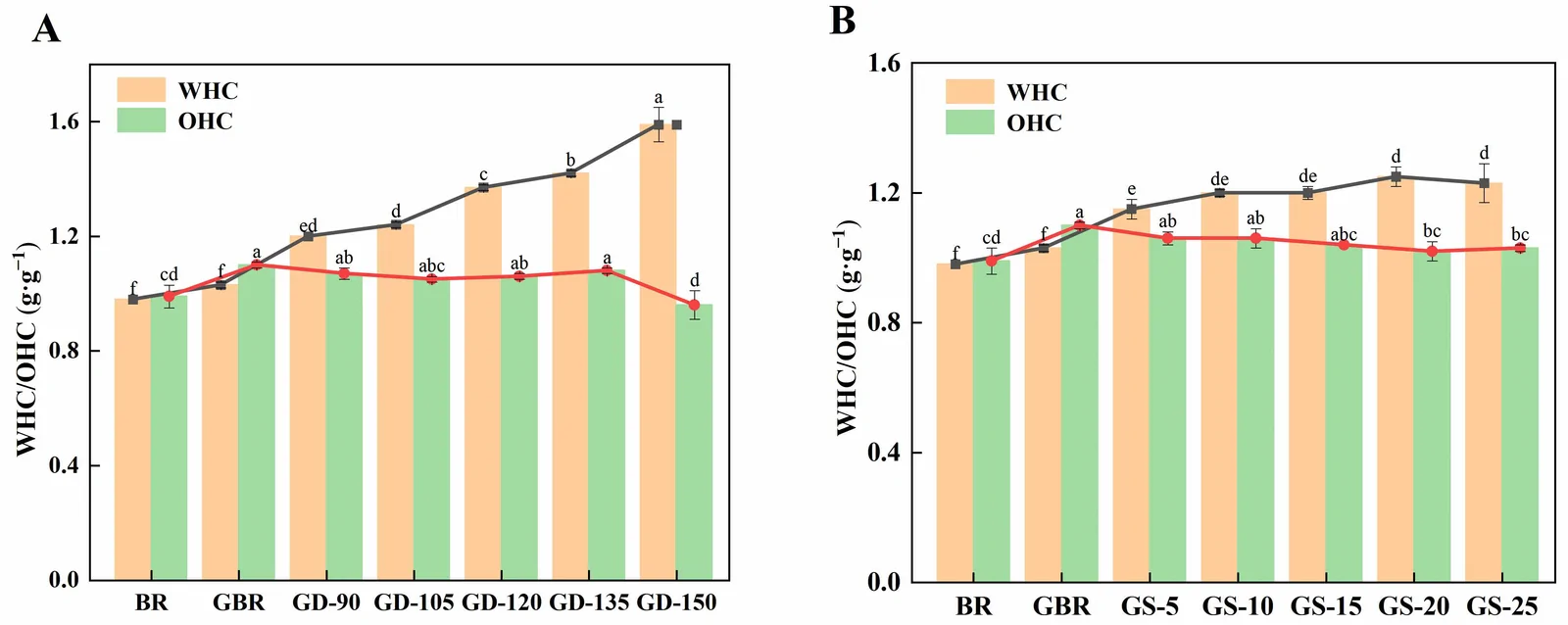
WHC and OHC of dry heat-treated (**A**) and high-pressure steam-treated (**B**) germinated brown rice flour. Orange bars with black square symbols denote water-holding capacity (WHC), and green bars with red circular dots denote oil-holding capacity (OHC). Different lowercase letters indicate significant differences among groups (*p* < 0.05).

WHC and OHC are important indicators for evaluating powder functional characteristics, reflecting the starch matrix’s ability to bind water and oil molecules, and are closely related to slurry network construction and 3D printing quality. As shown in Figure 4, after germination treatment, the WHC of GBR showed no significant difference compared with BR *(p* > 0.05). Further dry heat and high-pressure steam treatments of GBR significantly improved WHC as treatment intensity increased *(p* < 0.05). Compared to high-pressure steam treatment, dry heat treatment had a more significant effect on WHC *(p* < 0.05). High temperature breaks hydrogen bonds within starch molecules, exposing large amounts of hydrophilic hydroxyl groups and strengthening hydrogen bonds with water molecules. This effect was more pronounced under dry heat treatment, which induced greater structural damage to GBR starch. In terms of OHC, samples from all high-pressure steam treatment groups exhibited minor fluctuations, indicating that holding time had a limited effect on the powder’s OHC. For dry heat-treated samples, only the GD-150 group showed a significant reduction in OHC *(p* < 0.05). Excessively high temperatures caused excessive starch degradation, collapse of matrix pore structures, and reduction in oil adsorption sites, thereby reducing the powder’s OHC.

WHC affects the molding performance of 3D printing slurries. Moderately improving WHC helps build a continuous and stable gel network, enhancing the structural strength of the printed blank. If WHC is too high, it can easily cause excessive starch swelling, increased paste viscosity, and issues such as hindered extrusion and loose molded body structure. Overall, GD-120, GD-135, GS-10, and GS-15 samples had moderate WHC and stable OHC, and their corresponding pretreatment processes were suitable for preparing germinated brown rice 3D printing raw materials.

### 3.4. Variations in Pasting Properties

**Figure 5:**
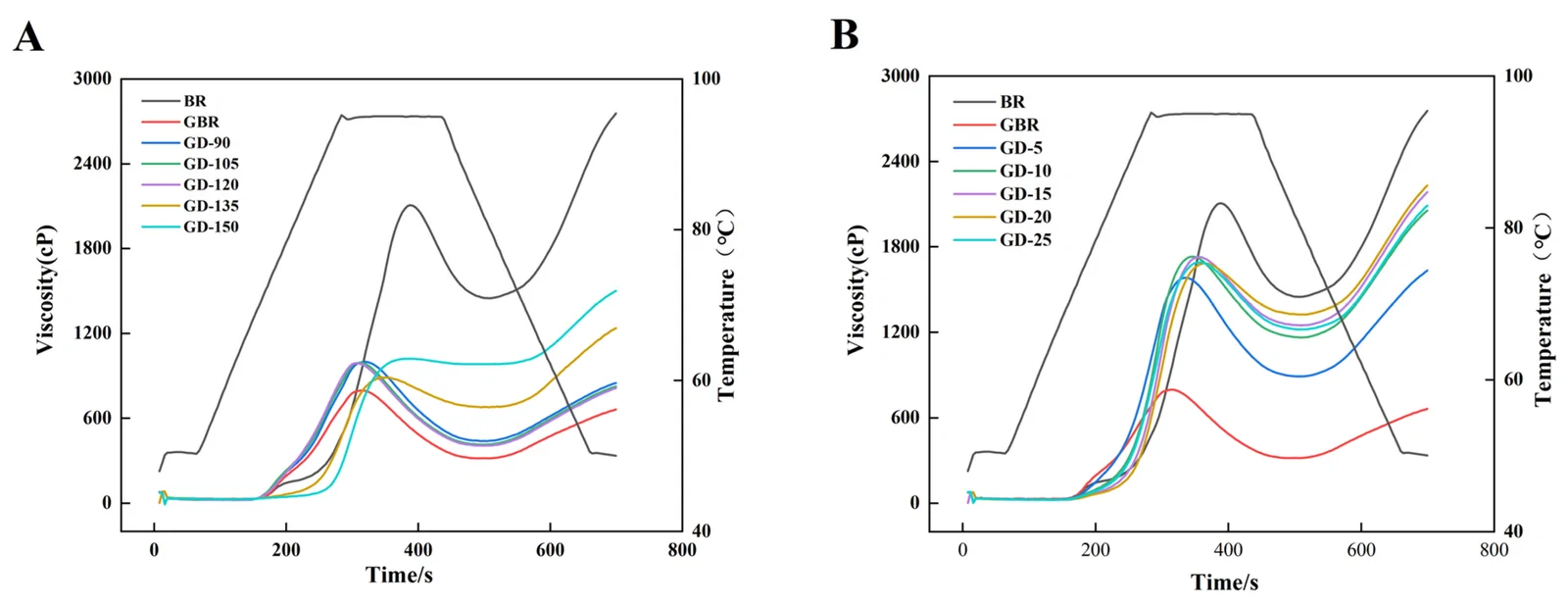
Pasting profiles of dry heat-treated (**A**) and high-pressure steam-treated (**B**) germinated brown rice flour.

Starch gelatinization is the process by which starch molecules break hydrogen bonds and their crystalline structure dissociates under hydrothermal conditions, causing the granules to absorb water, swell, and increase the viscosity of the system. The gelatinization and viscosity characteristics directly determine the rheological behavior and 3D printability of starch-based pastes [25]. The effects of dry heat and high-pressure steam modification on the RVA viscosity curves and characteristic parameters of germinated brown rice flour are shown in Figure 5 and Table 1, respectively. PV represents the maximum viscosity that starch granules can reach when fully swollen, which is an important indicator for evaluating paste extrusion. As seen in Table 1, compared to BR, germination significantly reduced the PV, TV, and FV of GBR (*p* < 0.05). This occurred mainly because germination activated endogenous starch enzymes, causing long-chain starch to degrade and the ordered structure of granules to be destroyed, weakening their swelling ability [16]. Both dry heat and high-pressure steam treatments effectively increased the viscosity of germinated brown rice flour, with overall recovery of PV, TV and FV. The viscosity parameters fell between those of BR and unmodified GBR. Compared with dry heat treatment, high-pressure steam treatment produced a significantly greater viscosity-enhancing effect *(p* < 0.05), and GS-10 reached a PV of 1751.50 cP. Hydrothermal conditions facilitated the repair of starch structural damage caused by germination, promoted the re-association of molecular chains, and restored starch pasting and swelling properties [26].

BD is a critical parameter for evaluating the thermal and shear stability of starch paste, where a lower BD value represents stronger resistance to structural breakdown under continuous heating and shearing conditions [12]. In this study, germination treatment significantly reduced the BD of brown rice starch *(p <* 0.05), suggesting that the germination process effectively improved the hot-paste stability of native rice starch. For dry heat-modified GBR samples, BD slightly increased from untreated GBR to GD-90 and reached the maximum at GD-105 (583.00 ± 5.66 cP), implying impaired hot-paste stability under low-temperature dry heat conditions. This phenomenon occurred because mild heat only partially disrupts starch particles, but it is insufficient to induce substantial molecular changes or intermolecular cross-linking, which reduces the thermal and mechanical resistance of starch. However, as dry heat temperature increased, BD decreased rapidly, and it reached an extremely low value for GD-135 (214.50 ± 4.95 cP) and GD-150 (39.50 ± 0.71 cP). A marked reduction in BD indicated superior hot-paste stability. Sufficient thermal treatment promoted extensive molecular cross-linking of starch, forming a compact and homogeneous gel network that maintained structural integrity during heating and stirring. For high-pressure steam samples, BD patterns were consistent: it peaked at GS-5 (698.00 ± 5.66 cP), then slowly dropped as the steam time increased, reaching the lowest at GS-15 (480.00 ± 2.83 cP) and GS-20 (363.50 ± 3.54 cP). Overall, GD-135, GD-150, GS-15, and GS-20 possessed the lowest BD magnitudes, confirming their superior thermal stability and shear resistance during paste processing.

SB characterizes the recrystallization and retrogradation behavior of starch paste during the cooling stage, and a lower SB corresponds to a weaker starch aging tendency. In the present study, thermal modification slightly increased the SB of GBR paste, whereas all treated groups still exhibited significantly lower SB values than raw BR *(p* < 0.05). Compared with high-pressure steam treatment, dry heat treatment was more effective in reducing SB, thereby conferring better cold-paste stability and slower retrogradation behavior on GBR starch. PV, BD, and SB are decisive rheological indicators for assessing the printability of starch-based printing inks. Appropriate peak viscosity, minimal hot-paste breakdown, and suppressed retrogradation behavior synergistically contribute to balanced rheological properties. Such optimized paste characteristics enable modified GBR samples to withstand continuous extrusion shear and post-printing structural stabilization, which greatly improves the processing adaptability and molding accuracy of germinated brown rice-based 3D printing materials.

### 3.5. Rheological Properties of 3D Food Ink

**Figure 6:**
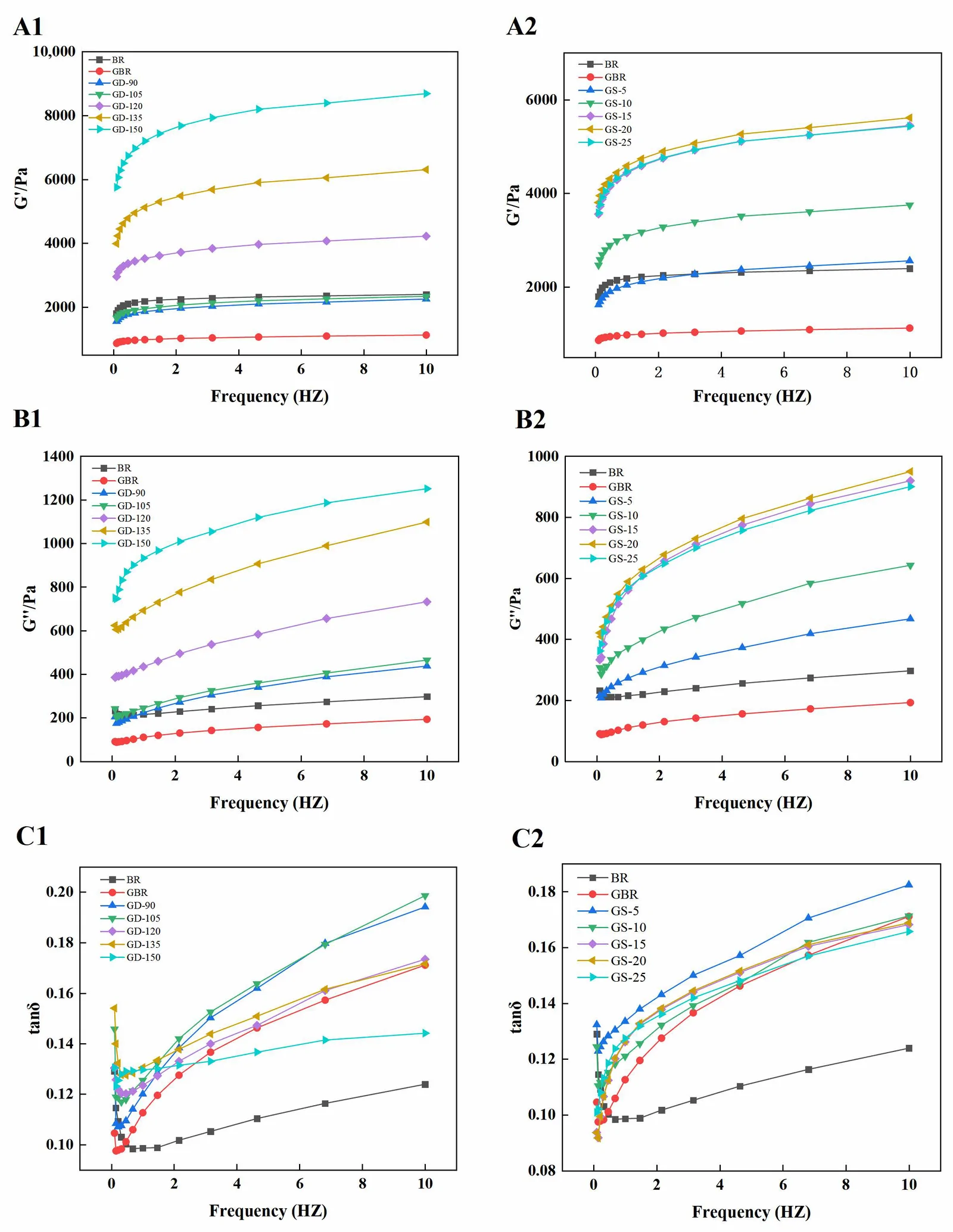
Frequency sweep of dry heat-treated (**A1**–**C1**) and high-pressure steam-treated (**A2**–**C2**) germinated brown rice flour pastes.

During 3D food printing, printable paste undergoes complex coupled pressure and shear stress fields, and its intrinsic rheological behaviors dominantly determine extrusion performance, structural recovery efficiency, and post-printing self-supporting capability [27]. The dynamic rheological characteristics of dry heat- and high-pressure steam-modified GBR gels were further investigated via frequency sweep tests, and the corresponding results were presented in Figure 6. Storage modulus (G′) and loss modulus (G″) represent the elastic and viscous components of viscoelastic gels, respectively [28], while the loss factor (tan δ) acts as a pivotal index to evaluate overall viscoelasticity. Basically, a lower tan δ value means the material is more elastic and can form gel networks that are stiffer and more stable [29].

As shown in Figure 6, as the oscillation frequency increased, G′ and G″ rose together. This occurred because higher oscillation frequencies intensify the friction between starch molecular chains, enhancing the viscoelastic response of the system. Germination treatment significantly lowered G’ and G″ of GBR compared to BR, as germination activated endogenous amylase, leading to the degradation of starch chains and a weaker gel network. After dry heat and high-pressure steam treatments, most modified germinated brown rice gels showed higher G’ than untreated brown rice and germinated brown rice samples. For 3D printing paste systems, higher G’ is key for supporting printed lines and improving shape retention, while moderate G″ ensures smooth extrusion during printing. In the dry heat treatment group, GD-90 and GD-105 only showed a slight increase in G’. In contrast, GD-120, GD-135, and GD-150 showed a noticeable increase in G’ and moderate G″, achieving a better balance between elasticity and extrusion flowability for the printing paste.

In addition, the tan δ values of all test samples were consistently below 1, indicating that elastic behavior dominated the viscoelastic system across the entire frequency scan range [30]. The tan δ of untreated BR and GBR, low-temperature dry heat groups (GD-90, GD-105), and short-time steam-treated samples (GS-5, GS-10) was relatively higher. This indicated a weaker gel network structure and poorer structural stability, making these pastes prone to collapse and shape deformation during 3D printing. In contrast, tan δ significantly decreased for GD-120, GD-135, GS-15, and subsequent treatment groups, demonstrating the predominance of elastic properties, which was favorable for maintaining shape after printing. However, excessive thermal treatment (GD-150, GS-25) continuously strengthened intermolecular interactions of starch chains, forming an overly rigid gel network and increasing the risk of filament breakage during extrusion. Overall, GD-120, GD-135, GS-10, and GS-15 exhibited the most balanced viscoelastic properties and are promising as precursor materials for 3D food printing.

### 3.6. Texture Profile Analysis

Texture characteristics play a key role in the sensory quality of food and consumer acceptance, rendering them an important evaluation indicator for the development and quality optimization of 3D-printed food materials. This study systematically analyzed the texture characteristics of GBR gels modified by dry heat treatment and high-pressure steam treatment, with the results summarized in Table 2. Hardness, as a core texture parameter, represents the gel samples’ ability to resist external compression and is highly related to the extrusion behavior and printability of 3D printing pastes. Excessively high hardness substantially increases extrusion resistance, which readily causes nozzle blockage and disrupts continuous printing [31]. Conversely, insufficient hardness weakens the self-supporting capability of extruded filaments, ultimately inducing structural collapse and morphological distortion of printed constructs. Therefore, moderate and tailored hardness is a prerequisite for achieving favorable shape fidelity and stable structural integrity of 3D-printed products [32].

As listed in Table 2, the hardness of native BR gel was 150.11 g, which significantly declined to 95.30 g after germination (*p* < 0.05). The germination process activated endogenous hydrolases that degraded starch granules and cleaved macromolecular chains, thereby loosening the original gel network structure and reducing gel hardness [33]. After dry heat modification, the hardness of GBR gels increased continuously and significantly as the treatment temperature increased from 90 °C to 150 °C (*p* < 0.05). For high-pressure steam treatment, gel hardness increased with the extension of holding time, while a slight reduction was observed when the treatment duration exceeded 15 min. Short-term hydrothermal stimulation facilitated the reassociation of degraded starch fragments to form a compact crosslinked network, which strengthened gel hardness. Nevertheless, excessive steam treatment triggered mild starch hydrolysis and network loosening, consequently inducing a slight decline in hardness.

Gumminess and chewiness correspond to the energy required to disintegrate gel matrices and form swallowable boluses during mastication, respectively. In this study, both gumminess and chewiness exhibited variation trends consistent with gel hardness. The above results further confirmed that heat modification promoted the reassembly and re-crosslinking of starch molecules in GBR gel, inducing moderate retrogradation, which ultimately helped form a denser, stiffer, and more stable gel network.

### 3.7. Moisture State Analysis

**Figure 7:**
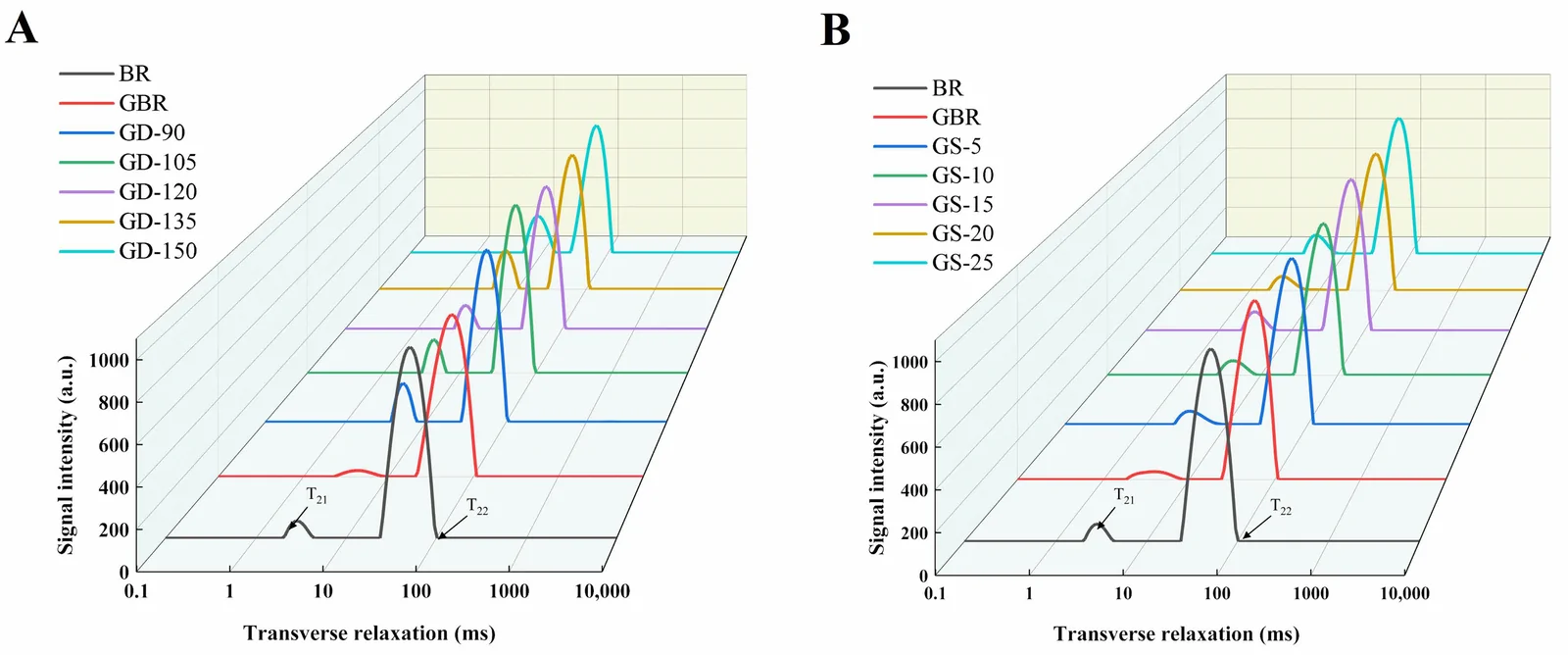
LF-NMR transverse relaxation time T_2_ distribution of dry heat-treated (**A**) and high-pressure steam-treated (**B**) germinated brown rice gels.

LF-NMR is a powerful non-destructive technique for characterizing the water state, spatial distribution, and molecular migration behavior within gel matrices. The existing forms and mobility of water are critical factors governing the rheological performance and 3D printability of starch-based materials [34]. In this study, LF-NMR transverse relaxation tests were performed to investigate the water dynamic characteristics of modified GBR gels, aiming to clarify the intrinsic differences in textural properties and printing accuracy from the perspective of water molecular mobility. Transverse relaxation time (T_2_) served as a reliable indicator to differentiate water fractions with distinct mobility, where a higher T_2_ value corresponded to stronger water mobility [2]. As shown in Figure 7, all the gel samples exhibited two distinct T2 relaxation peaks, representing two typical water components with different binding degrees. The short relaxation component T21 corresponded to strongly bound water, which was tightly attached to the starch chains through hydrogen bonds and had the lowest molecular mobility. The long relaxation component T22 represented water that was confined within the porous gel network and possessed medium molecular mobility [22]. Among the two water components, the T22 peak dominated the total peak area, indicating that less mobile water was the main form of water in the GBR gel system. Notably, no characteristic peak corresponding to free water was observed in any sample, suggesting that almost no free water existed in the prepared GBR gel matrix.

Compared with BR, the relaxation peaks of GBR obtained from germination shifted overall toward longer relaxation times, along with a decrease in the proportion of water peak area and increased mobility of water molecules. This occurred because germination activated endogenous amylase, degrading starch macromolecules, loosening the gel network structure, and weakening the water-binding capacity of the matrix. After dry heat and high-pressure steam treatments, as the treatment intensity increased, the relaxation peaks gradually shifted toward shorter relaxation times, the proportion of bound water corresponding to T21 steadily rose, and the mobility of water molecules became more restricted. Heat treatment promoted the reassociation of starch chains, forming a denser and more continuous gel network, exposing more hydroxyl sites, and enhancing the water-binding capacity of the matrix. This change in water distribution corresponded to the sample’s water retention tests, showing that a denser network simultaneously improved the gel’s water retention capacity. At the same treatment intensity, high-pressure steam treatment immobilized water molecules more effectively than dry heat treatment. An appropriate ratio of bound and semi-bound water in the system improved the self-supporting ability of printed pastes, effectively preventing water leakage-induced structural collapse and shape deformation.

### 3.8. FTIR Analysis

**Figure 8:**
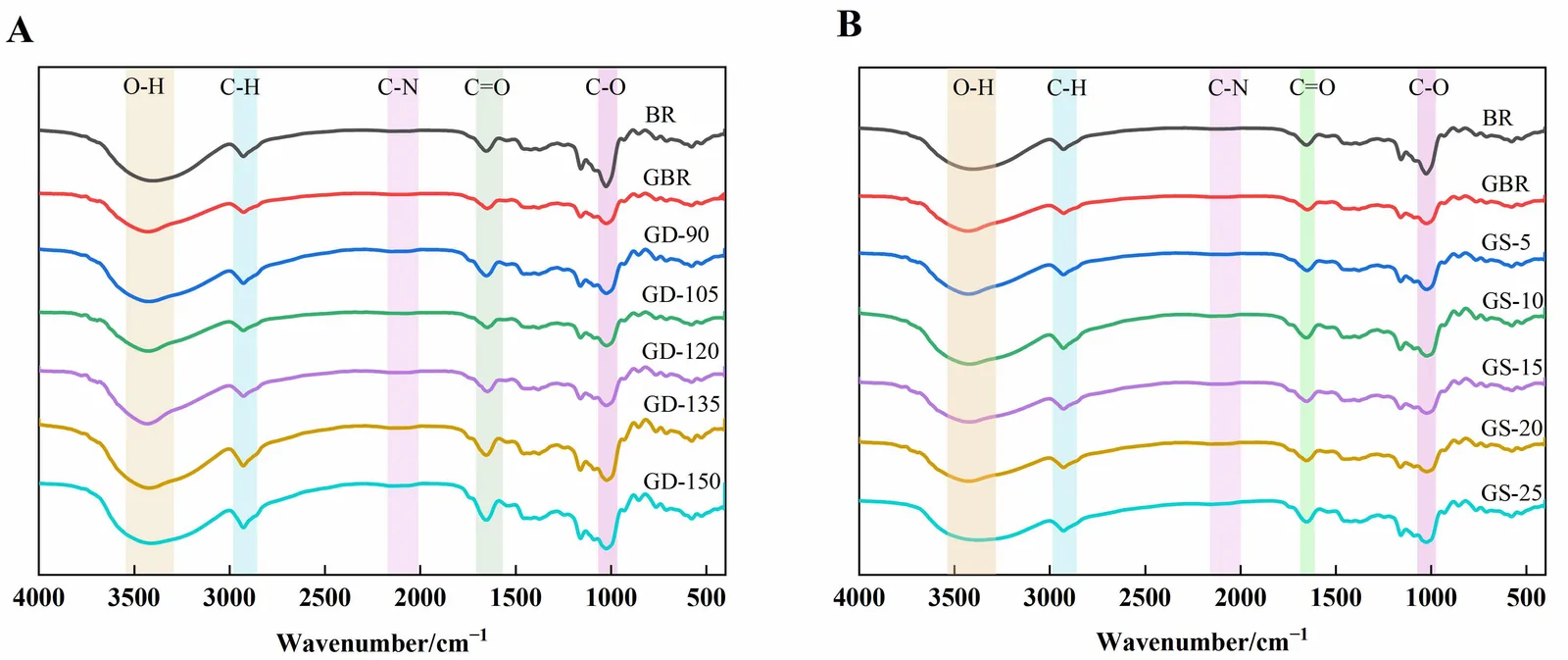
FT-IR spectra of dry heat-treated (**A**) and high-pressure steam-treated (**B**) germinated brown rice gels.

FT-IR spectra can characterize changes in starch functional group vibrations and intermolecular interactions caused by heat treatment [31]. As shown in Figure 8, the infrared absorption peak positions of BR, GBR, and all heat-treated samples were similar, with no new characteristic peaks appearing after thermal modification. The main absorption bands appeared around 3400, 2930, 1645, 1370, and 1022 cm^−1^. The broad absorption band near 3400 cm^−1^ is attributed to the O-H stretching vibration of hydroxyl groups; the peak at 1645 cm^−1^ originated from the amide I band of endogenous proteins and the H-O-H bending vibration of adsorbed water [2], reflecting the hydrogen bonding patterns and water-binding state inside the starch gel matrix. The absorption peaks of heat-modified germinated brown rice gel at 3400 cm^−1^ and 1645 cm^−1^ increased significantly, indicating that the intermolecular hydrogen bond network was effectively rebuilt and the water-holding capacity was enhanced. This structural change was consistent with the increase in bound water ratio observed in low-field NMR results. All samples showed stable C-H stretching vibration absorption peaks at 2930 cm^−1^, indicating that the basic carbon skeleton of the starch was not damaged and no molecular chain breakage occurred during processing [25]. The characteristic peak at 1022 cm^−1^ originated from the C-O stretching vibration of starch polysaccharides and was closely related to the short-range ordered structure of starch granules. Overall, FT-IR analysis confirmed that both dry heat treatment and high-pressure steam treatment were physical modification methods. These thermal treatments mainly regulated intermolecular interactions between starch molecules without changing the inherent functional groups or generating new chemical structures.

The spectral ratios R_1045_/_1022_ and R_1022_/_995_, quantitatively calculated from FT-IR spectra, served as reliable indicators for evaluating alterations in the short-range ordered structure and double-helix conformation of starch molecules. As presented in Table 3, germination, dry heat treatment, and high-pressure steam treatment remarkably decreased R_1022_/_995_ values compared with raw BR (*p* < 0.05), demonstrating partial unwinding and disruption of native starch double-helix structures. For dry heat-modified GBR samples, the R_1045_/_1022_ ratio increased steadily with elevated treatment temperature, indicative of gradual enhancement in the degree of short-range order. Similarly, high-pressure steam treatment improved the degree of order in starch structure, albeit less effectively than dry heat treatment. This difference is primarily ascribed to the water-plasticizing effect under hydrothermal conditions, which limits close rearrangement and aggregation of starch molecular chains [35]. Collectively, both thermal modification strategies disrupted inherent hydrogen bond networks in starch, reduced double-helix content, and increased the proportion of amorphous regions. These structural alterations effectively optimized extrusion fluidity of 3D printing pastes and enhanced the morphological and structural stability of GBR-based printed products.

### 3.9. XRD Crystallography

**Figure 9:**
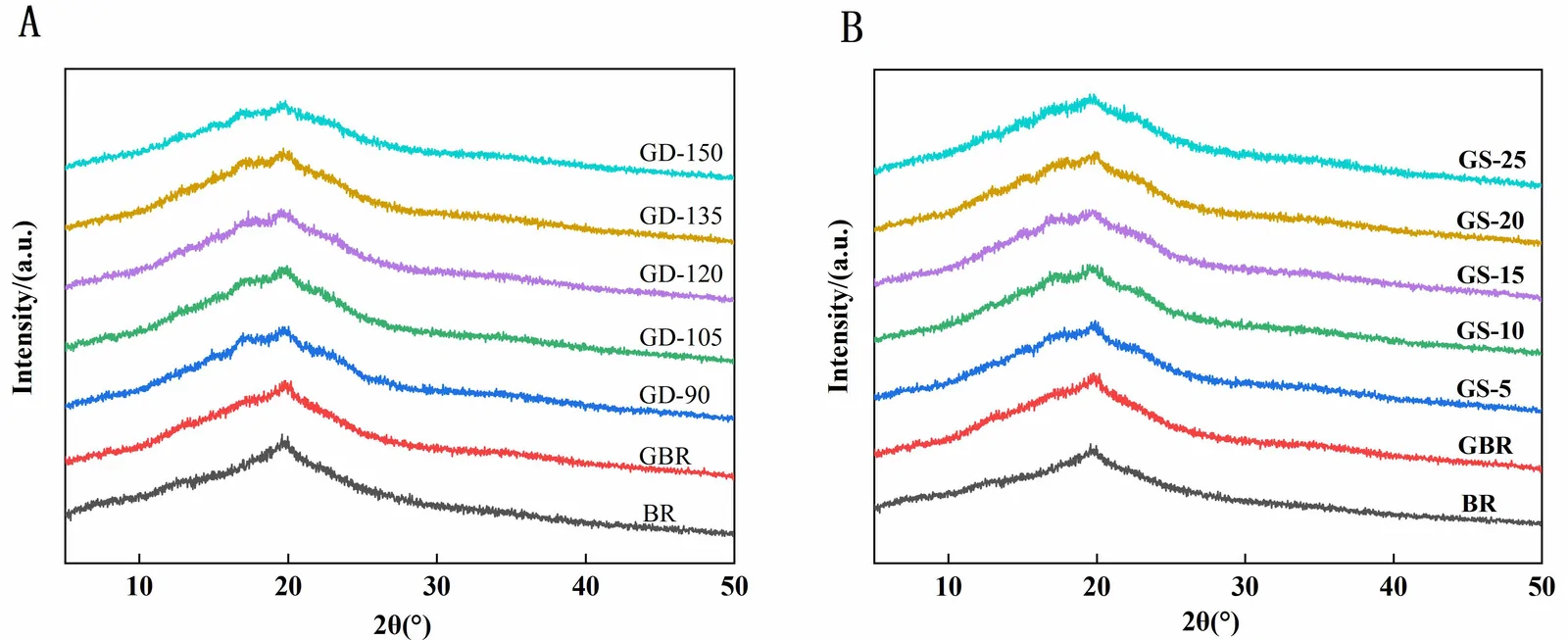
XRD patterns of dry heat-treated (**A**) and high-pressure steam-treated (**B**) germinated brown rice gels.

XRD is a pivotal technique for characterizing variations in the long-range ordered crystalline structure of starch materials [36]. Typically, A-type starch presents characteristic diffraction peaks at 2θ angles of 15°, 17°, 18°, 20°, and 23°, while B-type starch shows distinct diffraction signals at 5.6°, 15°, 17°, 20°, and 24°. As illustrated in Figure 9, native brown rice starch exhibited a typical A-type crystalline pattern, which was consistent with previous reports [37]. However, the inherent A-type crystalline structure was completely disrupted during gelatinization and gel formation. All prepared gel samples exhibited only a broad, diffuse diffraction peak at 2θ = 18–20°, demonstrating that the gel systems were predominantly composed of amorphous starch structures. Neither dry heat nor high-pressure steam treatment shifted the position of diffraction peaks, and no new crystal polymorph was induced. As the dry heat temperature increased and the high-pressure steam treatment time was extended, the diffraction peak intensity gradually strengthens, and the relative crystallinity also slightly increased. This finding was consistent with the previous FT-IR results. Heat treatment promoted the rearrangement and aggregation of starch molecular chains, enhancing both short-range and long-range order, but the crystal type did not change.

### 3.10. Morphology

#### 3.10.1. Microstructure of Heat-Treated Germinated Brown Rice Powders

**Figure 10:**
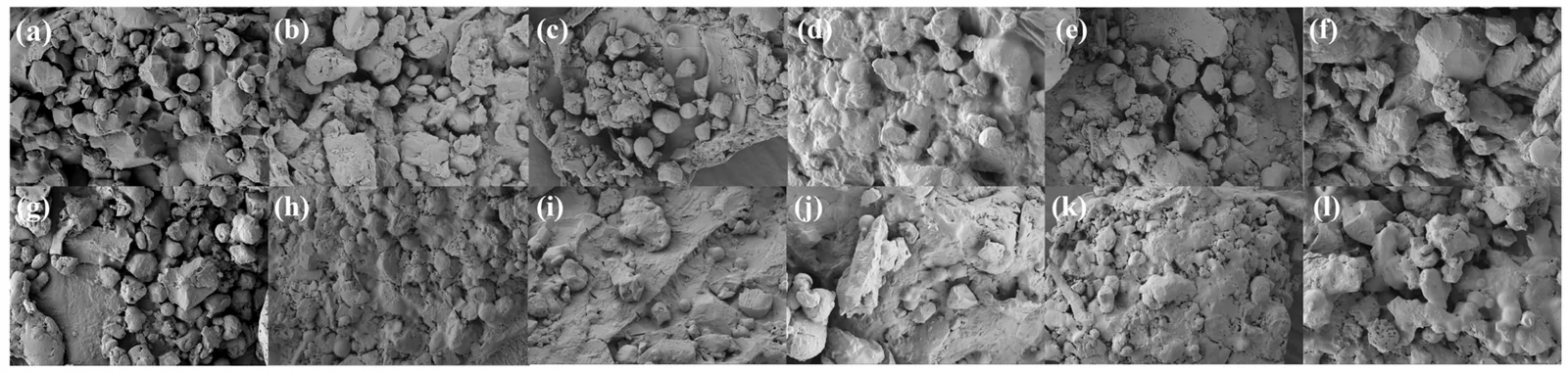
SEM images of germinated brown rice powders treated by dry heat and high-pressure steam (3000× magnification), where (**a**) is raw brown rice (BR), (**g**) is untreated germinated brown rice (GBR), (**b**–**f**) are dry heat treated samples at 90 °C, 105 °C, 120 °C, 135 °C and 150 °C, and (**h**–**l**) are high-pressure steam treated samples for 5 min, 10 min, 15 min, 20 min and 25 min, respectively.

SEM allows intuitive visualization of micromorphological variations in starch granules, providing direct evidence for structural changes induced by germination and thermal modification. As displayed in Figure 10, native BR starch granules presented irregular polygonal and quasi-spherical morphologies with complete outlines, clear boundary definition, and favorable particle dispersibility. The granule surfaces were smooth and structurally intact. Untreated GBR starch exhibited a granule morphology similar to that of BR, whereas slight inter-granule adhesion and aggregation were clearly observed. During germination, the activation of endogenous enzymes triggered partial degradation of starch macromolecules; the produced low-molecular-weight sugars and leached amylose subsequently promote granule agglomeration in GBR samples. Distinct micromorphological alterations were observed in GBR starch after dry heat and high-pressure steam treatments. All thermally modified samples retained the basic morphological outlines of native starch granules, while the degree of inter-granule aggregation increased substantially, with numerous individual granules tightly adhering to form large aggregated clusters. In addition, modified starch granules exhibited increased surface roughness with localized surface depressions and slight structural damage, and partial granule edges showed typical melting and adhesion characteristics. Comparative morphological analysis further demonstrated that high-pressure steam treatment induced more severe granule adhesion than dry heat treatment, accompanied by significantly blurred and diffuse granule boundaries.

#### 3.10.2. Microstructure of Heat-Treated Germinated Brown Rice Gels

**Figure 11:**
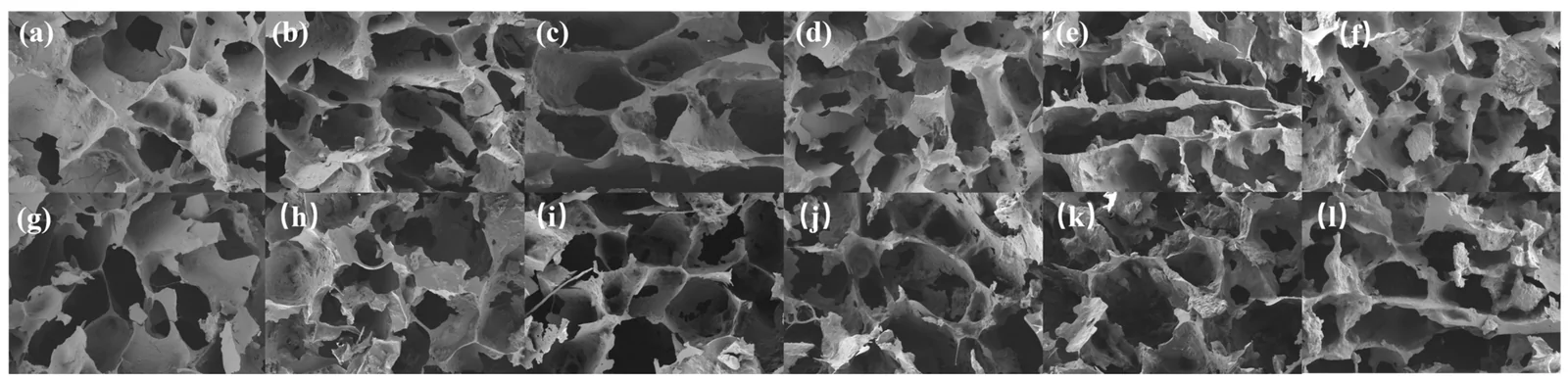
SEM images of 3D printed gels from germinated brown rice powders treated by dry heat and high-pressure steam (200 × magnification), where (**a**) is raw brown rice (BR), (**g**) is untreated germinated brown rice (GBR), (**b**–**f**) are dry heat-treated samples at 90 °C, 105 °C, 120 °C, 135 °C and 150 °C, and (**h**–**l**) are high-pressure steam-treated samples for 5 min, 10 min, 15 min, 20 min and 25 min, respectively.

Figure 11 further illustrates the microstructural differences in 3D-printed germinated brown rice gels modulated by dry heat and high-pressure steam treatments. The printed BR gel displayed a compact but disordered porous network structure. In comparison, enzymatic hydrolysis during germination induced partial degradation of starch macromolecules in untreated GBR, which loosened the integrity of the gel network, enlarged internal pores, and resulted in a highly heterogeneous pore size distribution.

After heat modification, the pore size of the gel network first decreased and then increased. This structural evolution mainly came from heat treatment causing starch molecules to rearrange and cross-link; the rearranged molecules then interact with the enzymatic products produced during germination, ultimately changing the way starch aggregates and the density of the gel network. Under moderate heat treatment, GBR gels form a uniform 3D porous structure, with suitable pore sizes, even distribution, and moderate wall thickness. The optimized dense network can effectively improve the mechanical strength and self-supporting ability of the starch gel, which helps achieve smooth extrusion, stable layer-by-layer deposition, and excellent shape retention during 3D printing. Nevertheless, excessive dry heat treatment (temperature exceeding 135 °C) caused severe rupture of starch molecular chains and impaired the structural-supporting capacity of the gel network, leading to pore re-expansion and compromised pore homogeneity. In comparison, high-pressure steam treatment provides a uniform heat and moisture environment, which helps the starch fully gelatinize and allows molecules to rearrange more orderly. Accordingly, compared to samples treated with dry heat, steam-modified gels exhibited more regular and uniform network microstructure. Overall, the changes in the gel’s microstructure matched well with the macroscopic printed shape. These results indicated that properly controlling the heat treatment method and intensity can effectively optimize the network structure of GBR gels, which is key for fabricating high-precision, well-structured 3D-printed whole-grain foods.

## 4. Conclusions

GBR powder was prepared through controlled germination and further modified using two green physical methods—dry heat treatment (90–150 °C) and high-pressure steam treatment (5–25 min). This study systematically investigated the effects of the two thermal treatments on the bioactivity, starch rheology, water migration, microstructure changes, and 3D printability of germinated brown rice. Results showed that controlled germination significantly degraded starch macromolecules, reducing the paste’s gelatinization viscosity, gel viscoelasticity, and self-supporting ability, ultimately inducing structural collapse and larger dimensional deviations in 3D printed products. In comparison, moderate dry heat and high-pressure steam treatments effectively increased the GABA content and noticeably improved the water-holding capacity of the powder. Proper heat regulation promoted the reorganization of starch molecules and intermolecular cross-linking, restoring the peak viscosity. Both the storage modulus and loss modulus (G′ and G″) increased; gel hardness also rose, and after optimizing the rheological and textural properties, the modified germinated brown rice paste could be extruded steadily, maintain favorable shape, and achieve higher printing precision.

Multi-scale structural characterization further elucidated the intrinsic modification mechanism. Dry heat treatment and high-pressure steam treatment mainly regulated the hydrogen bond interactions between molecules and did not generate new chemical functional groups. Heat induction partially unwinds the starch double-helix structure, enhancing the short-range order of starch molecules, thereby rebuilding a more stable gel network. Scanning electron microscopy confirmed that the moderately treated groups (GD-120, GD-135, GS-15) formed a uniform three-dimensional porous structure, with appropriately sized pores and continuous, intact pore walls. The optimized physicochemical properties and uniform microstructure collectively accounted for the favorable printability of the modified germinated brown rice material. Notably, GD-135 and GS-15 exhibited well-defined layered printing morphologies, minimal dimensional deviation, and complete structural outlines, which were identified as the optimal pretreatment conditions for high-precision 3D food printing.

In summary, targeted moderate thermal modification synchronously enhances the nutritional functionality and 3D printability of germinated brown rice. The core optimization mechanism lies in the regulation of starch molecular aggregation behavior, water-binding capacity and distribution, and gel micro-network architecture. This study clarifies the structure–rheology–printability correlation of thermally modified whole-grain starch systems and provides a feasible theoretical basis and technical reference for the development of green, chemical-free modification strategies for high-performance 3D-printed whole-grain food raw materials.

## Figures and Tables

**Table 1:** Pasting properties of germinated brown rice flour under different treatments.

	PV/cP	TV/cP	BD/cP	FV/cP	SB/cP	PT/min	Ptemp/°C
BR	2130 ± 30 ^a^	1456 ± 10 ^a^	670 ± 10 ^a^	2963 ± 4 ^a^	1507 ± 9 ^a^	6.43 ± 0.05 ^a^	88.4 ± 0.6 ^b^
GBR	800 ± 10 ^g^	322 ± 9 ^i^	482 ± 2 ^c^	740 ± 20 ^j^	416 ± 8 ^h^	5.27 ± 0.00 ^f^	73.0 ± 0.6 ^g^
GD-90	1010 ± 10 ^e^	440 ± 4 ^h^	567 ± 8 ^b^	935 ± 8 ^h^	416 ± 8 ^g^	5.30 ± 0.05 ^f^	72.1 ± 0.53 ^g^
GD-105	998 ± 9 ^e^	415 ± 4 ^h^	583 ± 6 ^b^	906 ± 6 ^hi^	496 ± 5 ^g^	5.20 ± 0.00 ^f^	72.4 ± 0.5 ^g^
GD-120	980 ± 20 ^e^	403 ± 2 ^h^	570 ± 10 ^b^	880 ± 10 ^i^	480 ± 10 ^g^	5.07 ± 0.00 ^g^	73.0 ± 0.6 ^g^
GD-135	903 ± 20 ^f^	690 ± 20 ^g^	215 ± 5 ^e^	1350 ± 20 ^g^	660 ± 30 ^e^	5.80 ± 0.09 ^d^	87.7 ± 0.6 ^bc^
GD-150	1010 ± 20 ^e^	970 ± 20 ^e^	39.5 ± 0.7 ^f^	1590 ± 40 ^f^	620 ± 20 ^f^	6.40 ± 0.09 ^a^	91.7 ± 0.5 ^a^
GS-5	1590 ± 10 ^d^	894 ± 8 ^f^	698 ± 6 ^a^	1794 ± 8 ^e^	900.0 ± 0.7 ^d^	5.60 ± 0.00 ^e^	76.3 ± 0.6 ^f^
GS-10	1750 ± 30 ^b^	1180 ± 20 ^d^	577 ± 10 ^b^	2240 ± 10 ^d^	1070 ± 5 ^c^	5.77 ± 0.05 ^d^	83.6 ± 0.5 ^e^
GS-15	1731 ± 2 ^b^	1251 ± 5 ^c^	480 ± 3 ^c^	2381 ± 4 ^c^	1130 ± 1 ^b^	5.93 ± 0.00 ^c^	85.6 ± 0.07 ^d^
GS-20	1670 ± 20 ^c^	1310 ± 20 ^b^	364 ± 4 ^d^	2430 ± 10 ^b^	1119 ± 7 ^b^	6.07 ± 0.00 ^b^	87.20 ± 0.00 ^c^
GS-25	1690 ± 1 ^c^	1190 ± 50 ^d^	500 ± 40 ^c^	2260 ± 40 ^d^	1070 ± 10 ^c^	5.93 ± 0.09 ^c^	84.00 ± 0.00 ^e^

Note: All values are expressed as mean ± standard deviation. Different lowercase letters in the same column indicate significant differences (*p* < 0.05).

**Table 2:** Textural properties of germinated brown rice gels subjected to dry heat and high-pressure steam treatments.

	Hardness (g)	Springiness (mm)	Cohesiveness	Gumminess (g)	Chewiness (g)	Resilience
BR	150 ± 1 ^d^	0.95 ± 0.01 ^a^	0.72 ± 0.00 ^b^	108.3 ± 0.7 ^hi^	103 ± 2 ^ef^	0.08 ± 0.01 ^bc^
GBR	95.3 ± 0.4 ^f^	0.95 ± 0.01 ^a^	0.76 ± 0.05 ^ab^	73 ± 6 ^j^	69 ± 6 ^g^	0.07 ± 0.00 ^c^
GD-90	103 ± 2 ^ef^	0.95 ± 0.01 ^a^	0.75 ± 0.03 ^ab^	77 ± 4 ^j^	73 ± 5 ^g^	0.10 ± 0.00 ^ab^
GD-105	119 ± 4 ^e^	0.95 ± 0.01 ^a^	0.76 ± 0.02 ^ab^	89.7 ± 0.3 ^ij^	85 ± 2 ^fg^	0.09 ± 0.01 ^bc^
GD-120	141 ± 4 ^d^	0.96 ± 0.01 ^a^	0.77 ± 0.02 ^ab^	150 ± 10 ^de^	140 ± 20 ^cd^	0.10 ± 0.01 ^ab^
GD-135	251 ± 5 ^b^	0.94 ± 0.01 ^a^	0.77 ± 0.08 ^ab^	190 ± 20 ^b^	180 ± 20 ^b^	0.10 ± 0.00 ^ab^
GD-150	310 ± 10 ^a^	0.95 ± 0.00 ^a^	0.77 ± 0.01 ^ab^	240 ± 20 ^a^	230 ± 20 ^a^	0.12 ± 0.02 ^a^
GS-5	150 ± 10 ^d^	0.95 ± 0.01 ^a^	0.78 ± 0.00 ^ab^	120 ± 8 ^g^	114 ± 5 ^e^	0.08 ± 0.00 ^bc^
GS-10	170 ± 20 ^d^	0.95 ± 0.01 ^a^	0.78 ± 0.00 ^ab^	130 ± 10 ^ef^	120 ± 10 ^de^	0.09 ± 0.01 ^bc^
GS-15	223 ± 3 ^d^	0.95 ± 0.03 ^a^	0.75 ± 0.02 ^ab^	130 ± 10 ^ef^	120 ± 10 ^de^	0.09 ± 0.01 ^bc^
GS-20	220 ± 9 ^c^	0.93 ± 0.00 ^a^	0.76 ± 0.01 ^ab^	167 ± 3 ^cd^	155 ± 3 ^c^	0.09 ± 0.01 ^bc^
GS-25	218 ± 2 ^c^	0.94 ± 0.01 ^a^	0.81 ± 0.01 ^a^	176 ± 5 ^bc^	165 ± 2 ^bc^	0.09 ± 0.00 ^bc^

Note: All values are expressed as mean ± standard deviation. Different lowercase letters in the same column indicate significant differences (*p* < 0.05).

**Table 3:** FT-IR spectral intensity ratios R1045/1022 and R1022/995 of germinated brown rice gels with dry heat and high-pressure steam treatments.

	R1045/1022	R1022/995
BR	0.89 ± 0.00 ^i^	1.20 ± 0.00 ^a^
GBR	0.92 ± 0.00 ^g^	1.11 ± 0.00 ^b^
GD-90	0.94 ± 0.00 ^e^	1.08 ± 0.00 ^e^
GD-105	0.91 ± 0.00 ^h^	1.09 ± 0.00 ^c^
GD-120	0.93 ± 0.00 ^f^	1.08 ± 0.00 ^de^
GD-135	0.93 ± 0.00 ^f^	1.05 ± 0.00 ^f^
GD-150	1.10 ± 0.00 ^a^	0.92 ± 0.01 ^h^
GS-5	0.92 ± 0.00 ^g^	1.09 ± 0.00 ^cd^
GS-10	0.94 ± 0.00 ^cd^	1.04 ± 0.00 ^g^
GS-15	0.94 ± 0.00 ^d^	1.06 ± 0.00 ^f^
GS-20	0.95 ± 0.00 ^c^	1.06 ± 0.00 ^f^
GS-25	0.95 ± 0.00 ^b^	1.08 ± 0.00 ^de^

Note: Data are expressed as mean ± standard deviation. Different superscript letters in the same column indicate significant differences at (*p* < 0.05).

## Data Availability

The original contributions presented in the study are included in the article, further inquiries can be directed to the corresponding author.
